# Do Indonesian medical practitioners approve the availability of emergency contraception over-the-counter? A survey of general practitioners and obstetricians in Jakarta

**DOI:** 10.1186/1472-6874-5-3

**Published:** 2005-03-22

**Authors:** Dyna E Syahlul, Lisa H Amir

**Affiliations:** 1 Key Centre for Women's Health in Society University of Melbourne, Australia; 2 Faculty of Medicine, Universitas Indonesia, Indonesia

## Abstract

**Background:**

Few studies have examined the attitude of medical practitioners towards the availability of emergency contraception (EC) without prescription. In Indonesia, EC (either Yuzpe regimen or Postinor-2) is available by prescription only. We aimed to examine the level of knowledge, attitudes and practices of medical practitioners in Indonesia about EC, in particular their attitudes to the availability of EC over-the-counter (OTC), using a questionnaire.

**Methods:**

Data were collected by an anonymous structured questionnaire. Questionnaires were distributed to general practitioners in 36 Community Health Centres and 25 private clinics using stratified random sampling according to area in Jakarta, and to obstetricians practicising in 24 government and private hospitals and eight private clinics in Jakarta. Two hundred and five general practitioners and 142 obstetricians and gynaecologists participated; overall response rate was 75%.

**Results:**

Although most participants were familiar with EC, only 22% received a very good knowledge score (4 or 5/5 answers correct), while 52% received a poor score (0–2/5 correct). Most participants did not support the OTC availability of EC (70%). Logistic regression identified that participants who prescribed EC had an Odds of 3.8 (95% CI 1.90, 7.73) of approving OTC EC, after adjustment for age and speciality.

**Conclusion:**

Although many organisations are working towards OTC availability of EC, it needs to be recognized and addressed that doctors who do not prescribe EC are unlikely to support the increased availability of EC.

## Background

Family planning practitioners and women's health advocates have been campaigning for increased availability of emergency contraception (EC) [1-4]. Grimes and colleagues have stated that EC meets all the criteria for over-the-counter (OTC) use such as low toxicity, no potential for overdose or addiction, no teratogenicity, no need for medical screening, self-identification of need, uniform dosage, and no important drug interactions [2]. In many countries EC is now available OTC from pharmacies, after a consultation with a trained pharmacist, but without a doctor's prescription [5].

The progestogen-only regimen, using two 0.75 mg levonorgestrel tablets either together or 12 hours apart, is more effective than the Yuzpe regimen in preventing pregnancy (RR 0.51; 95% CI 0.31, 0.83) [6] and is available pre-packaged as Postinor-2 or Plan B [3,4].

Only a small number of studies have examined the attitude of medical practitioners towards the availability of EC without prescription. Although Postinor-2 is available OTC in Vietnam, the majority of family planning workers participating in focus groups favoured availability by doctor's prescription [7]. Most health care providers interviewed in Mexico City (n = 40) favoured distribution of EC through hospitals and clinics [8]. Interviews with family planning providers in Kenya found a mixed response to distribution of EC; some providers expressing concern that pharmacists may not be able to monitor the distribution as well as family planning clinics [9]. Other studies also found that a low proportion of health care providers believed EC should be available through pharmacies: nine percent of health care providers surveyed in Nigeria (n = 735) [10] and 22% of GPs and O&Gs in India (n = 130) [11]. Although only 14% of practitioners surveyed in Minnesota, USA (n = 495), supported OTC availability of EC, 63% of physicians approved a trained pharmacist providing EC after conducting a focused history, making appropriate referrals to a physician for STD screening, sexual assault counselling, physical exam, etc (if indicated) [12].

In Indonesia, EC is available by prescription only, either the Yuzpe regimen (eg 2 Neogynon pills, repeated in 12 hours) or progestogen-only (Postinor-2) [13].

This study aimed to examine the level of knowledge, attitudes and practices of medical practitioners in Indonesia about EC, in particular their attitudes to the OTC availability of EC. The only study of providers' knowledge and attitudes to EC in Indonesia which we identified is an unpublished study by Lubis and colleagues cited by the Consortium for Emergency Contraception [14]. Only 25% of health care providers (GPs, O&Gs, nurse-midwives) and policy makers (family planning program managers, members of professional associations, Ministry of Health officials, and religious and community leaders) were familiar with EC [14].

## Methods

### Study subjects

The study participants were medical practitioners in Jakarta, Indonesia. The researchers aimed to distribute questionnaires to 210 GPs and 250 obstetricians and gynaecologists (O&Gs). In Indonesia patients may self-refer to obstetricians and gynaecologists.

The Provincial Health Service-Jakarta (Suku Dinas Kesehatan DKI Jakarta) provided a list of Community Health Centres (CHCs) (Pusat Kesehatan Masyarakat (Puskesmas)) and private clinics in five municipalities in Jakarta. Thirty six CHCs and 25 private clinics were chosen using stratified random sampling according to area, from the total of 328 CHCs at district and village levels and 63 private clinics in Jakarta. A research assistant distributed the questionnaires to the CHCs and clinics along with a culturally-appropriate token of appreciation (souvenir pen), and later collected the completed questionnaires.

The Department of Obstetrics and Gynaecology, Faculty of Medicine, the University of Indonesia, Ciptomangunkusumo's Hospital (Bagian Obstetri dan Ginekologi, Fakultas Kedokteran-Universitas Indonesia, Rumah Sakit Ciptomangunkusumo) approved the study and distributed the questionnaires to O&Gs practicing in 24 government and private hospitals and eight private clinics in Jakarta.

### Survey instrument

Data were collected by an anonymous structured questionnaire consisting of 30 questions. The first section collected data about the socio-demographic and work characteristics of the participants. The second section assessed the knowledge, attitudes and practices of the participants about EC. Participants were asked if they had ever heard of the terms "emergency contraception", "morning-after pill" or "post coital contraception" and if they were familiar with the specially packaged emergency contraceptive pill, Postinor-2. Knowledge about Postinor-2 was assessed using five statements, with the responses "true", "false" or "not sure". Five statements regarding participants' attitudes towards the availability of EC as an OTC product were provided with a 5-point Likert scale. In addition, two case scenarios were provided to indicate circumstances in which participants would prescribe EC to their patients, with 5-point Likert scale responses.

The final item on the questionnaire was an invitation for participants to make free- text comments: "We are interested in your thoughts about emergency contraception, please write any comments here". After pilotting with a small number of Australian and Indonesian doctors and medical students, some minor alterations were made to the survey.

Translated questionnaires were copied in Melbourne and sent to Indonesia for distribution. Participants were given the questionnaire along with a Plain Language Statement and a letter of a support from either the GP or O&G organisation, as appropriate. Data collection took place in February and March 2004, and completed questionnaires were couriered back to Melbourne in April 2004 for data entry and analysis.

### Analysis

EpiData 3.0 [15]was used for data entry and Epi Info Version 3.2 [16] and Stata 8.0 [17] were used for analysis. Frequency and summary statistics were calculated for all variables. Relevant variables were cross-tabulated and Wilcoxon-Mann-Whitney and Kruskall-Wallis tests were conducted to explore the association between the variables. A logistic regression model was developed to look at the factors predictive of acceptance of the OTC availability of EC (dependent variable). Independent variables were speciality, gender, age, marital status, religion, knowledge score, oral contraceptive pill prescriber and EC prescriber. Stata 8 was used for this analysis.

Participants' comments were translated and typed in English. Selected comments were used to support and illustrate the main themes emerging from participants' responses. This study was approved by the Human Research Ethics Committee, The University of Melbourne.

## Results

Two hundred and five of the 210 (97.6%) general practitioners (GPs) and 142 of the 250 obstetricians and gynaecologists (O&Gs) (56.8%) completed the questionnaire, yielding an overall response rate of 75% (347/460). Some participants completed the demographic responses fully, but did not complete all the responses about emergency contraception, therefore denominators for responses vary.

Demographic and work characteristics of participants are presented in Table 1. Fifty four percent of participants were male, with a mean age of 42 years (range 24–68). Most participants were married (82%) and the dominant religious affiliation was Islam (68%). Almost all participants worked in Jakarta, the capital city of Indonesia (93%).

**Table 1:** Demographic and clinical work characteristics of participants

**Characteristics**	**GP (n = 205)**		**O&G (n = 142)**		**Total (n = 347)**	
	**n**	**%**	**n**	**%**	**n**	**%**
**Gender**						
Male	80	39	108	76	188	54
Female	125	61	34	24	159	46

**Age**						
Mean age (years old)	39		45		42	
Younger	121	59	51	36	172	50
Older	83	41	91	64	174	50
Missing	1				1	

**Current martial status**						
Single	55	27	1	1	56	16
Married	146	71	137	96	283	82
Divorced			3	2	3	1
Widow/ widower	4	2	1	1	5	1

**Religion**						
Islam	108	53	127	89	235	68
Catholic	39	19	5	4	44	13
Protestant	48	24	9	6	57	16
Hindu	1	0	1	1	2	1
Buddhist	7	3			7	2
Other	1	0			1	0
Missing	1				1	

**Location of work**						
Jakarta	187	92	131	94	318	93
Other	3	1	6	4	9	3
Both	13	6	2	1	15	4
Missing	2		3		5	

**Main place of work**						
Government hospital			73	58	73	23
Private hospital	5	3	38	30	43	14
Government clinic	2	1			2	1
Private clinic	80	43	15	12	95	30
Community health centre	99	53			99	32
(*PUSKESMAS*)	1	1			1	0
Other	18		16		34	

**Provide advice on contraception in clinical work**						
Yes	191	94	139	99	330	96
No	12	6	2	1	14	4
Missing	2		1		3	

**Prescribed OC in the last six months**						
Yes	133	66	126	89	259	75
No	70	34	15	11	85	25
Missing	2		1		3	

All percentages are calculated on valid response only

GP: general practitioner, O&G: obstetrician and gynaecologist, OC: oral contraception

^1^Younger: 21 – 40 years, older: 41 – 70 years

The majority of participants provided advice on contraception in their practices (GP 94%; O&G 99%). A higher proportion of O&Gs had prescribed oral contraception (OC) than GPs in the six months prior to the survey (O&G 89%; GP 66%).

More O&Gs had heard of the term "emergency contraception (EC)"or "morning-after pill" or "post coital contraception" or "special pill preventing pregnancy" (O&G 130/141, 92%; GP 148/203, 73%). More than half the GPs who answered this question were familiar with the progestogen-only pill (POP) as an EC method (89/139, 64%). The two most common EC methods mentioned by O&Gs were POP and the Yuzpe regimen (POP 88/127, 69%; the Yuzpe regimen 82/127, 65%). Fifty two percent of GPs (78/150) and 66% of O&Gs (85/128) had heard of Postinor-2 specifically. Participants reported hearing about Postinor-2 from lectures, meetings or seminars (83/273, 30%), journal articles (83/273, 30%) and their colleagues (69/273, 25%).

The results of the five knowledge questions are presented in Table 2. Knowledge scores were regarded as very good (4 or 5/5 correct), good (3/5 correct) or poor (0, 1 or 2/5 correct). Only 22% of participants received a very good knowledge score, while 52% received a poor score.

**Table 2:** Knowledge about progestogen-only emergency contraception

	**GP (n = 151)**		**O&G (n = 131)**		**Total (n = 282)**	
	**n (m)^♣^**	**%**	**n (m)^♣^**	**%**	**n (m)^♣^**	**%**
**Correct responses to questions about POP, Postinor-2**						
Dosage: 0.7 5 mg levonorgestrel × 2 (T)	54 (12)	39	71 (10)	59	125 (22)	48
Timing of administration: within 84 hrs (F)	51 (8)	36	48 (8)	39	99 (16)	37
Causes abortion (F)	49 (8)	34	77 (7)	62	126 (15)	47
Fewer side-effects than Yuzpe regimen (T)	27 (7)	19	59 (8)	48	86 (15)	32
Efficacy 85% (T)	99 (7)	69	98 (7)	79	197 (14)	74

**Knowledge score**						
Very good (4 or 5 out of 5 correct)	10	7	46	39	56	22
Good (3 out of 5 correct)	39	28	28	24	67	26
Poor (0 or 1 or 2 correct)	90	65	45	38	135	52
Missing	12		12		24	

All percentages are calculated on valid response only

GP: general practitioner, O&G: obstetrician and gynaecologist, POP: progestogen-only pill, T: true, F: false

♣ The numbers in brackets are the missing values

Seventy percent (193/276) of the participants disapproved of the availability of EC as an over the counter (OTC) product (GP 120/149, 81%; O&G 73/127, 57%). Fourteen responded that they were "not sure".

Two case scenarios were provided to indicate circumstances in which participants would prescribe EC to their patients. The majority of participants would prescribe EC in the case of contraceptive failure in a married couple (GP 105/146, 72%; O&G 112/130, 86%). Similarly, most participants would also provide EC to a young woman who had been raped (GP 97/146, 66%; O&G 118/130, 91%).

More O&Gs reported prescribing EC than GPs in the six months before this survey (O&G 83/126, 66%; GP 74/144, 51%). An equal proportion of GPs and O&Gs had provided the Yuzpe regimen in their practices (GP 54/144, 38%; O&G 49/129, 38%). In addition, 37% (48/129) of O&Gs had also prescribed POP as EC (GP 28/144, 19%). About half the GPs had never prescribed EC in their clinical practices (GP 70/144, 49%; O&G 43/129, 33%). The IUD was also used more by O&Gs than GPs (O&G 18/119, 15%; GP 4/131, 3%).

Participants were dichotomized into two age groups (aged 40 years and below, and over 40 years). Older participants were more knowledgeable about EC than younger participants (z score = 2.071; *p *= 0.0384). However, stratification by speciality resulted in no significant association between level of knowledge and age group (GP, chi-square = 3.0993, *p *= 0.212; O&G, chi-square = 0.0620, *p *= 0.969). There was a significant difference in the knowledge level between GPs and O&Gs (z score = 5.486; *p *< 0.0001), with O&Gs having higher knowledge scores.

Participants who prescribed oral contraception (OC) in the six months before the survey were more likely to have a "very good" level of knowledge, especially about Postinor-2, than participants who had not prescribed OC (z score = 3.381; *p *= 0.0007). The difference in the level of knowledge about EC was not significantly related to gender (z score = 1.765; *p *= 0.0775) or location of work (chi-square with ties = 4.035; *p *= 0.2577).

Table 3 presents the association between approval of OTC availability of EC and age, gender, speciality, location of work, knowledge score and prescribing EC. More male participants accepted the OTC status of EC (z score = 2.036; *p *= 0.0418). O&Gs were more likely to accept EC as an OTC product than GPs (z score = 3.410; *p *= 0.0006). More older participants approved the OTC status of EC product than younger participants (z score = 3.029; *p *= 0.0025). Most participants disapproved the availability of EC OTC, however participants with "very good" and "good" knowledge score were more likely to approve the OTC status of EC than participants with "poor" knowledge (z score = 2.626; *p *= 0.0086). However, stratification of attitude by speciality showed that significant difference was only found among GPs (GP, chi-square = 11.7983, *p *= 0.003; O&G, chi-square = 0.8995, *p *= 0.638).

**Table 3:** The association between approval for OTC availability of EC and other variables

**Variables (n = 276)**	**Approves EC as an OTC product**		**Disapproves EC as an OTC product^1^**		
	**n**	**%**	**n**	**%**	
**Age group^2^**					
Younger	23	18	121	82	0.0025^4^
Older	43	34	85	66	
Missing = 1					
**Gender**					
Male	47	30	112	70	0.0418
Female	22	19	95	81	
**Speciality**					
GP	25	17	124	83	0.0006
O&G	44	35	83	65	
**Location of work**					
Jakarta	63	25	190	75	
Other	1	14	6	86	0.8569
Both	4	31	9	69	
Missing = 3					
**Knowledge score^3^**					
Very good	19	34	37	66	
Good	22	33	45	67	0.0086
Poor	25	19	110	82	
Missing = 18					
**Prescribes EC**					
Yes	57	37	96	63	<0.0001
No	12	11	100	89	
Missing = 5					

EC: emergency contraception; GP: general practitioner, O&G: obstetrician and gynaecologist, OTC: over the counter

^1^Includes "not sure"

^2^Younger: 21 – 40 years, older: 41 – 70 years

^3^Very good (4 or 5 out of 5 correct), Good (3 out of 5 correct), and Poor (none or 1 or 2 out of 5 correct)

^4^Wilcoxon-Mann-Whitney test was used to examine the association between variables and attitude (Kruskall-Wallis test was used for location of work)

The EC practices were different across age group, gender, speciality, knowledge and attitude scores. Older GPs were more likely to prescribe EC in their practices (older 69/112, 62%; younger 75/157, 48%; z score = 2.239; *p *= 0.0252). Male participants provided EC more commonly than females (male 105/156, 67%; female 52/114, 46%; z score = 3.562; *p *= 0.0004). In addition, being an O&G was a positive predictor for prescribing EC (O&G 83/126, 66%; GP 74/144, 51%; z score = 2.402; *p *= 0.0163). Participants who had better knowledge about EC were more likely to provide EC (z score = 3.378; *p *= 0.0007). However, stratification of EC practice by speciality showed a significant relationship between level of knowledge and prescribing EC only for GPs (GP, Pearson chi-square = 6.6889, *p *= 0.035; P&G, Pearson chi-square = 3.4616, *p *= 0.177). Participants who accepted the OTC status of EC were more likely to use EC in their clinical work (accepted OTC status 57/69, 83%; disapproved OTC status 96/196, 49%; z score = 4.854; *p *< 0.0001).

A logistic regression model was developed to look at the factors predictive of acceptance of the over-the-counter availability of emergency contraception. The independent variables (speciality, gender, age, marital status, religion, knowledge score, OC prescriber and EC prescriber) were tested individually against the dependent variable using logistic regression to determine Odds Ratios. The p-values of the Wald statistic for all variables were <0.05, except religion which was 0.179 and marital which 0.693. Independent variables were entered in the model if the p-value of the Wald statistic was = 0.25 [18], therefore all variables were entered initially except marital status. From the stratified univariate analysis, it was recognized that age was a confounding factor as it was associated with speciality; therefore both were retained in the model. Records with missing data on any of these variables were removed from the sample, leaving 247 records for analysis.

Variables were eliminated one at a time using logistic regression, only those with a p-value of the Wald statistic of ≤ 0.05 were retained in the model [18]. The process was repeated until only significant variables remained. Then the marital variable was added back into the model to check that if it was now significant given the reduced model. Interactions were examined between age and speciality and age and sex. These were not retained in the model as the Wald statistic p-values were > 0.01 (p = 0.623 and 0.525 respectively).

To check how closely aligned the predicted and observed data values were, a 'goodness of fit' test was performed (Hosmer-Lemeshow chi2(6) = 1.54, p = 0.9565); the non-significant p value indicates a good fit. The sensitivity of the model, that is, how often it correctly predicted the outcome (y) given the value of a value of a covariate (x) was tested using the area under the ROC curve. To be said to have good discrimination, a model should have a statistical value of = 0.7 [18]. The lroc test identified that the area under ROC curve was 0.7195. The lstat test showed 72.03% correctly classified. The final model, with Odds Ratios, can be seen in Table 4.

**Table 4:** Approval for OTC availability of EC

		**Unadjusted**	**Multivariate analysis**
		**Odds Ratio (95%CI)**	**Adjusted Odds Ratio (95%CI)**
	
**EC prescriber**	No	Reference	Reference
	Yes	4.35 (2.18, 8.68)	3.70 (1.83, 7.50)
**Age (yrs)**	21–40	Reference	Reference
	41–70	2.66 (1.49, 4.76)	1.96 (1.05, 3.66)
**Speciality**	GP	Reference	Reference
	O&G	2.37 (1.33, 4.23)	1.76 (0.94, 3.28)

EC: emergency contraception; GP: general practitioner, O&G: obstetrician and gynaecologist

Many participants made comments about the use of EC in the final section of the questionnaire. Some participants stressed the emergency nature of this method:

• Agree on providing contraception/emergency contraceptive pill which might be used only in the case of contraceptive failure (forgetting to take regular oral contraceptive pill or condom leakage) (46 year-old male O&G)

Some participants argued that EC would be beneficial to reduce the abortion rate and prevent unwanted pregnancy.

• Emergency contraception is very useful for people wanting to prevent pregnancy. (It) may reduce the abortion rate as well as the complications of abortion (42 year-old female GP)

Participants had concerns about the availability of EC OTC in encouraging intercourse among unmarried couples especially adolescents.

• Do not market emergency contraception without prescription because may lead to free sex among teenagers which later causes STD, for example HIV AIDS (50 year-old male GP)

Most participants indicated that EC should be available by prescription.

• Emergency contraception should be given with prescription, not to be sold as an over the counter drug! (35 year-old female O&G)

Another theme was the need for more information about EC for both professionals:

• Need symposium for doctors, midwives, health personnel (62 year-old male O&G)

and the community:

• Need more explanation/ information for public through printed mass media/ TV/ radio (62 year-old male O&G).

## Discussion

This survey of general practitioners (GPs) and obstetricians and gynaecologists (O&Gs) in Indonesia has identified a low level of knowledge about emergency contraception (EC). While the majority of medical practitioners supported the availability and the use of EC, they disapproved the availability of EC as an over the counter (OTC) product. This survey also revealed that EC was prescribed infrequently by Indonesian medical practitioners.

The higher response rate among GPs, 98%, compared to O&Gs, 57%, was likely to be due to the different method of recruitment. The research assistant visited GPs' workplaces and gave them a small token (pen), while O&Gs received the questionnaire through their medical organization, without any contact with the researchers. However, although demographic questions were well answered, many participants did not complete all the questions; questions relating to prescribing practices were completed by 70% of GPs (144/205).

In our study, O&Gs were more likely to be involved in contraceptive care than GPs, and to be more familiar with the concept of EC. Although O&Gs were more likely to approve EC as an OTC product on univariate analysis, this was not significant on multivariate analysis. Logistic regression identified that participants who prescribed EC had an Odds of 3.7 of approving over-the-counter EC after adjusting for age and speciality.

There is a common misconception that EC is an abortifacient. Twenty percent of family planning coordinators in Michigan, USA, believed that EC worked by causing an abortion [19]. Anna Glasier states "It cannot be stressed too strongly that if hormonal emergency contraception works largely by interfering with ovulation, then it cannot be regarded as an abortifacient" [1 p1063].

A survey involving 100 O&Gs in Massachusetts, United States revealed that 94% had prescribed EC, with 57% reported prescribing it no more than five times per year [20]. In contrast, only 76% of 96 family physicians reported prescribing EC, and 82% of them prescribed it five times or less [20]. Lower levels of prescribing have been found in studies in developing countries. In Nairobi, Kenya, only 15% of family planning providers reported prescribing EC [9] and only 20% of primary health care workers recommended EC in Turkey [21]. In our survey, 66% of O&Gs and 51% of GPs reported prescribing EC in the previous six months, but as many participants did not complete this question, this should be regarded as an overestimate of prescribing practices.

A survey of family physicians and nurses (n = 78) in the USA found that practitioners who prescribed EC were more likely to agree that the benefits outweigh the risks (94%) than nonprescribers (50%) [22]. All of the nonprescribers (n = 7) agreed that they felt uncomfortable prescribing EC for religious / ethical reasons, compared to 8% of the prescribers [22].

The low level of support for OTC availability of EC found in our study is similar to the results of other studies [7,10,11]. A study in Oxford, UK, in which 76 GPs were interviewed in 1996, found that only eight of the GPs reported unqualified enthusiasm for deregulation of EC [23]. The four main themes expressed by the GPs were that women would be missing out on the benefits of the consultation; that EC is not the most effective, or safe, form of contraception; that some women may use it frequently; and that the pharmacy has characteristics which make it unsuitable for dispensing EC [23]. Ziebland has suggested that health professionals may feel uncomfortable with EC, because in contrast to other methods of contraception, the provision of EC is closely associated with a sexual episode [24].

## Conclusion

Medical practitioners in Indonesia would benefit from additional education about EC. Medical practitioners should be encouraged to include discussion of EC in their routine consultations with women about contraception. Although many organisations are working towards OTC availability of EC, it needs to be recognized and addressed that doctors who do not prescribe EC are unlikely to support the increased availability of EC.

## Competing interests

The author(s) declare that they have no competing interests.

## Authors' contributions

Both authors participated in the design, conduct and analysis of the study. Both authors read and approved the final manuscript.

## Pre-publication history

The pre-publication history for this paper can be accessed here:


